# Counting Mycobacteria in Infected Human Cells and Mouse Tissue: A Comparison between qPCR and CFU

**DOI:** 10.1371/journal.pone.0034931

**Published:** 2012-04-19

**Authors:** Sharad Pathak, Jane A. Awuh, Nils Anders Leversen, Trude H. Flo, Birgitta Åsjø

**Affiliations:** 1 Section for Microbiology and Immunology, The Gade Institute, University of Bergen, Bergen, Norway; 2 Department of Microbiology and Immunology, Haukeland University Hospital, Bergen, Norway; 3 Department of Cancer Research and Molecular Medicine, Norwegian University of Science and Technology, Trondheim, Norway; University of Padova, Italy

## Abstract

Due to the slow growth rate and pathogenicity of mycobacteria, enumeration by traditional reference methods like colony counting is notoriously time-consuming, inconvenient and biohazardous. Thus, novel methods that rapidly and reliably quantify mycobacteria are warranted in experimental models to facilitate basic research, development of vaccines and anti-mycobacterial drugs. In this study we have developed quantitative polymerase chain reaction (qPCR) assays for simultaneous quantification of mycobacterial and host DNA in infected human macrophage cultures and in mouse tissues. The qPCR method cannot discriminate live from dead bacteria and found a 10- to 100-fold excess of mycobacterial genomes, relative to colony formation. However, good linear correlations were observed between viable colony counts and qPCR results from infected macrophage cultures (Pearson correlation coefficient [r] for *M. tuberculosis* = 0.82; *M. a. avium* = 0.95; *M. a. paratuberculosis* = 0.91). Regression models that predict colony counts from qPCR data in infected macrophages were validated empirically and showed a high degree of agreement with observed counts. Similar correlation results were also obtained in liver and spleen homogenates of *M. a. avium* infected mice, although the correlations were distinct for the early phase (<day 9 post-infection) and later phase (≥day 20 post-infection) liver r = 0.94 and r = 0.91; spleen r = 0.91 and r = 0.87, respectively. Interestingly, in the mouse model the number of live bacteria as determined by colony counts constituted a much higher proportion of the total genomic qPCR count in the early phase (geometric mean ratio of 0.37 and 0.34 in spleen and liver, respectively), as compared to later phase of infection (geometric mean ratio of 0.01 in both spleen and liver). Overall, qPCR methods offer advantages in biosafety, time-saving, assay range and reproducibility compared to colony counting. Additionally, the duplex format allows enumeration of bacteria per host cell, an advantage in experiments where variable cell death can give misleading colony counts.

## Introduction

Rapid methods for enumerating slow-growing mycobacteria like *M. tuberculosis*, *M. avium avium* and *M. avium paratuberculosis* in cell cultures or infected tissues are needed both in human and veterinary medicine. Traditionally, quantification of mycobacteria is done by seeding serial dilutions of bacterial suspensions on suitable media such as Middlebrook 7H10 agar or Lowenstein Jensen followed by counting colony-forming units (CFU). However, this method is hampered by the long generation time and the tendency of mycobacteria to aggregate, resulting in multiple founders of a single colony and an underestimation of the correct number of bacteria. Typically, the time required for visible colonies to appear on 7H10 agar is 2–3 weeks for *M. tuberculosis* and *M. a. avium*, while it takes about 4–8 weeks for *M. a. paratuberculosis*. In addition, plating enough dilutions to make sure the results can be reliably counted is a tedious task that gives piles of plates with biohazardous bacteria. A further disadvantage of the colony counting method is that it cannot be reliably conducted on frozen samples, which may be both more practical and desirable in several research settings. Another method often used for quantification of mycobacteria is the radiometric measurement of the growth index (GI) using the BACTEC system. Though more rapid than colony counting, it still requires 1–4 days. In addition, the GI is not only dependent on the initial bacterial number but is also influenced by the replication characteristics of the particular bacterium and the metabolic rate. Hence, the GI of different mycobacterial species is not directly comparable, and bacteria grown under different experimental conditions may display different levels of dormancy making GI comparisons problematic.

We have previously validated a real-time quantitative PCR assay (qPCR) for detection of mycobacteria in a diagnostic setting [1]. Several other studies have also described distinct qPCR assays for detection and quantification of specific mycobacteria in a clinical setting and PCR based methods are now well established for mycobacterial diagnostics in both human and veterinary medicine. However, to our knowledge surprisingly few studies have directly compared qPCR with CFU counting in research settings [2], [3]. In this paper, we have refined our qPCR assay into a duplex format that allows simultaneous quantification of both mycobacterial and mammalian host DNA (human or mice). We have further compared the performance of the qPCR assay to traditional CFU counting in infected macrophage cultures with or without HIV co-infection. Mycobacterial infection in the context of HIV is a clinical problem. It is therefore important that experimental methods may accommodate for this scenario. We also compared the performance of the qPCR assay to traditional CFU counting in mouse tissue.

## Materials and Methods

### Ethics Statement

This research used peripheral blood from 15 anonymous HIV negative blood donors obtained from the Bloodbank at Bergen Hospital Trust, Haukeland University Hospital, Bergen, Norway. Blood donors give a general consent for the use of their blood for research at the time of donation; as such no further informed consent was required as all data were analyzed anonymously.

All experiments involving mice were kept to a minimum and performed according to protocols approved by the local Animal Care Committee (ref. 40/05, St Olavs hospital, Norway).

### Mycobacterial strains

Frozen aliquots with predetermined CFU of the clinical isolate S72/89 (serovar 4) of *M. a. avium* obtained from the blood of an AIDS patient (kindly provided by Dr. Sven Hoffner, Dept. of Bacteriology/TB section, Swedish Institute for Infectious disease Control, Solna, Sweden) and of the human clinical isolate Linda of *M. a. paratuberculosis* (kindly provided by Dr. Ingrid Olsen, Dept. of Animal Health, National Veterinary Institute, Oslo, Norway) were used in this study. The *M. tuberculosis* H37Rv strain (ATCC no. 27294, kind gift from Dr Harleen Grewal, The Gade Institute, University of Bergen, Bergen, Norway) was grown in Middlebrook 7H9 broth (Difco/Becton Dickinson) with OADC enrichment (Difco/BD) and 0.05%Tween 80, passed five times through a sterile syringe with a 29G needle to disrupt bacterial clumps before aliquots were frozen at −80°C and CFU determined by plating appropriate dilutions on Middlebrook 7H10 agar (Difco/BD). Virulent *M. a. avium* clone 104 expressing firefly luciferase (kind gift from David R. Sherman [4]) was cultured in Middlebrook 7H9 broth supplemented with glycerol, Tween 80 and ADC.

### Human monocyte-derived macrophages

Peripheral blood mononuclear cells (PBMC) were isolated from buffy coats of 15 anonymous HIV negative blood donors (from the Bloodbank at Bergen Hospital Trust, Haukeland University Hospital, Bergen, Norway) by centrifugation on a lymphoprep (AXIS-SHIELD PoC AS, Norway) density gradient as previously described [5]. For differentiation into monocyte-derived macrophages (MDM), PBMCs were cultured in RPMI 1640 supplemented with glutamine and 20% pooled heat-inactivated human serum (HS) for 5 days, followed by washing and further maturation for 2 more days in RPMI/10% HS as previously described [6].

### *In vitro* infection of macrophages

Seven days post isolation half of the MDMs from each donor were infected with HIV-1 BaL at a viral titer corresponding to 5 ng/ml of p24 in cell culture medium and incubated overnight at 37°C in 5%CO_2_. Culture medium was changed the next day and the HIV infection was further allowed to establish itself for seven days before mycobacterial infection. Two weeks old MDMs with or without HIV infection were incubated overnight at 37°C in 5%CO_2_ with the specified mycobacteria at an estimated multiplicity of infection (MOI) of 5 CFU per cell. In order to remove extra-cellular bacteria, cells were washed three times with PBS. (The assessment of the effect of HIV on mycobacterial growth has been published separately [6]). Cells infected with *M. tuberculosis* H37Rv or *M. a. avium* (S72/89) were cultured for one week, while cells infected with *M. a. paratuberculosis* were cultured for 14 days. Infected cells from at least three parallel wells of each donor were lysed in sterile water at specified time points and appropriate serial dilutions seeded on Middlebrook 7H10 agar plates in, at least, duplicates. Aliquots of the same lysates were also stored in cryotubes containing 250 µl of acid washed glass beads ≤106 µm in size (Sigma-Aldrich, Norway) at −80°C for later DNA extraction and use in qPCR assays.

### *In vivo* mouse infection

Infection was done as described previously [7] using log phase *M. a. avium* clone 104 expressing firefly luciferase (1–5×10^7^ bacteria/mouse) in 0.5 ml PBS, by peritoneal injection into 8–12 weeks old C56Bl/6 mice (Bomholdt, Denmark). The inoculum was plated on 7H10 agar plates for determination of the injected dose. Organs (spleen and liver) were harvested from 4–5 mice at given times post infection and homogenized in PBS/0.05% Tween80 using an Omni tissue homogenizer with a 10×95 mm saw tooth stainless steel generator probe (Omni International, GA, US). Organ homogenates were plated in serial dilutions for colony counting, or quickly frozen in liquid nitrogen for 10–15 min and stored at −80°C until use, usually within 1 to 3 weeks, for quantification by qPCR.

### DNA extraction for qPCR

#### From *in vitro* infected macrophages

Mycobacteria were inactivated by boiling cryotubes containing lysate and micro glass beads in a water bath for 20 min. Subsequently, the mycobacteria were disrupted mechanically by bead beating using a Ribolyser (Hybaid, UK) at max speed (6.5 m/s) for 45 s. The resulting crude mycobacterial DNA extract was used directly in the qPCR assay without further purification.

#### From mouse tissues

Frozen samples of organ homogenates were thawed on ice and then aliquoted into tubes containing about 0.5 g of 0.5 mm glass beads (VWR International, USA). Mycobacteria were inactivated by heating at 95°C in a heat block for 20 min. Mycobacteria were further disrupted mechanically by bead-beating using Precellys 24 bench-top lysis and homogenization equipment (Bertin Technologies, France) 3 times at a speed of 6700 rpm for 30 s. DNA extraction was done using the MasterPure Complete DNA and RNA Purification Kit from EPICENTRE Biotechnologies, USA, according to the manufacturer's protocol for extraction of total DNA from fluids.

### Quantitative real-time PCR

#### From *in vitro* infected macrophages

A duplex quantitative real-time PCR assay was set up for the human *β-globulin* gene and the mycobacterial heat shock protein 65 gene *GroEL2* using the QuantiTect Multiplex PCR Kit (Cat. No 204543, QIAGEN,West Sussex, UK) as per the manufacturer's instructions for TaqMan Probes with a final concentration of all primers and probes of 0,4 µM and 0,2 µM, respectively. For the human *β-globulin* gene, a 150 bp long segment was amplified using the primers BG-F (5′-TGCCTATCAGAAAGTGGTGGCT-3′) and BG-R (5′-GCTCAAGGCCCTTCATAATATCC-3′) and the probe BG-TAQ (5′TGGCTAATGCCCTGGCCCACAA-3′) as previously described [8]. However, due to the need for multiplexing and optimal performance, a TaqMan-MGB probe tagged with FAM was used instead of the FAM and TAMRA tagged TaqMan probe originally described [8]. For the mycobacterial *GroEL2* gene, a 103 bp long segment was amplified using the primers MycoFP1 (5′-CGAGGCGATGGACAAGGT-3′) and TB 12 (5′-CTTGTCGAACCGCATACCCT-3′) with the VIC tagged TaqMan-MGB probe MycoPr1 (5′-AACGAGGGCGTCATCACCGTCG-3′) as previously published by our group [1]. The qPCR was performed with a 7500 Fast-Real-Time System (Applied Biosystems, Foster City, CA, USA) with the following thermal cycling conditions: 95°C for 15 min to activate HotStartTaq DNA Polymerase, followed by 40 cycles of 94°C for 1 min and 60°C for 1 min. Standard curves were included in each qPCR run and were generated from known dilutions of commercially available genomic human male DNA (Applied Biosystems) and genomic *M. tuberculosis* H37Rv DNA available through TB Vaccine Testing and Research Materials Contract (Mycobacteria Research Laboratories, Colorado State University, Colorado, USA). The amount of DNA in each human or mycobacterial DNA standard was converted to number of human targets or mycobacterial targets by simply dividing with the estimated molecular weight of the haploid human genome (3.3 pg) or mycobacterial genome (4.8 fg) as each amplicon is a single-copy element in the respective genome.

#### From mouse tissues

Similarly, a duplex quantitative PCR was performed on DNA extracts from infected mouse tissue homogenates. The mycobacterial heat shock protein 65 gene *GroEL2* was amplified with the same primers and probe as above using the TaqMan Universal PCR Master Mix (Applied Biosystems, CA, USA). A 62 bp long segment of the murine *peptidylprolyl isomerase A (ppia)* gene was also amplified in the duplex reaction. The custom-designed primers for *ppia* were as follows; forward, 5′-GAGCCACTCACCTGATGCTTA-3′, reverse, 5′-GGCAATGAAAATGCTACCACCTT-3′ and the FAM-tagged MGB probe 5′-ACCTGTCAGCATAGCTT-3′. All primers and probes were used at a concentration of 8 µM and 4 µM, respectively. The qPCR was performed in the Applied Biosystems StepOnePlus™ Real Time PCR system, as follows: precycling steps of 50°C for 2 min and then 95°C for 10 min followed by 40 cycles of 95°C for 1 min and 60°C for 1 min. Standard curves were included in each qPCR run and were generated from known dilutions of mouse genomic DNA (The Jackson Laboratory, Maine, USA) and genomic *Mycobacterium sp* (LGC Standards, VA, USA).

### Statistical analysis

All data were analyzed using the SPSS version 15.0 statistical software package for Windows (SPSS Inc., Chicago, IL, USA).

#### Data from *in vitro* infected macrophages

All the paired CFU and qPCR values were log transformed. Each data set from macrophage cell cultures infected with the different mycobacterial strains was then split into two subsets using the random case selection option in SPSS set at 50%. All data from one of the subsets for each mycobacterial strain were used to calculate the Pearson correlation coefficient for investigation of an association and regressed to generate a prediction model for log(CFU) based on log(qPCR). Subsequently, the performance of each prediction model was tested in the appropriate second data subset by performing an agreement analysis of the predicted log(CFU) versus the observed log(CFU) using the Bland and Altman approach [9].

#### Data from mouse tissues

The paired CFU and qPCR data were log transformed and the Pearson correlation coefficient calculated. As the distribution of CFU to qPCR ratios were skewed, the ratios were log transformed to render the data normally distributed. The difference between the mean log transformed ratios of early versus later time-points was analyzed by an independent samples *t*-test and the result reported as the back transformed geometric means.

## Results

### Assay Performance: Dynamic range and Reliability of qPCR assays

Key performance characteristics of the mycobacterial assay are summarized in Table 1. In preliminary experiments using ten-fold serial dilutions of genomic *M. tuberculosis* H37Rv DNA with known concentration, we established that the qPCR for mycobacterial *GroEL2* gene has a sensitivity of detection corresponding to one mycobacterial genome (approximately 5 fg of genomic DNA) and a very wide dynamic range from 10^0^–10^7^ targets per reaction.

**Table 1:** Key Characteristics of the Mycobacterial *GroEL2 gene* qPCR Assay.

Factor	Mycobacterial assay
**Dynamic Range**	**10^0^–10^7^ Target Molecules**
**Sensitivity of Detection**	**1 Target copy** (5 fg of mycobacterial DNA)
**Mean slope of 5 replicate standard curves (±2× SD)**	**−3.37 (±0.12)**
**PCR Efficiency (±2× SD)**	**98.0% (±5.0%)**
**Coefficient of Determination (R^2^)**	
→Mean R^2^ of 5 replicate standard curves (±2× SD)	**0.991 (±0.008)**
**Intra-run Variation**	
→Estimated Range in % of Target Molecules from 5 replicate runs	**±3.55% to ±6.25%.**
**Inter-run Variation**	
→Mean % of Target Molecules (range) from 5 replicate runs	**4.80% (±0.07 to ±15.35)**

The number of bacteria and human cells usually used in macrophage virulence assays are in the range of 10^5^–10^7^ mycobacteria and 10^4^–10^6^ adherent human macrophages per well. Under our experimental conditions, this corresponded to an intermediate range of 500 to 40 000 mycobacterial *GroEL2* targets per reaction (final PCR mixture volume of 20 µl per reaction) and a low range of 50 to 1000 host *β-globulin* targets per reaction. Hence, the variability in the assays was examined over these ranges using serial dilutions of mycobacterial and human DNA, respectively.

**Figure 1:**
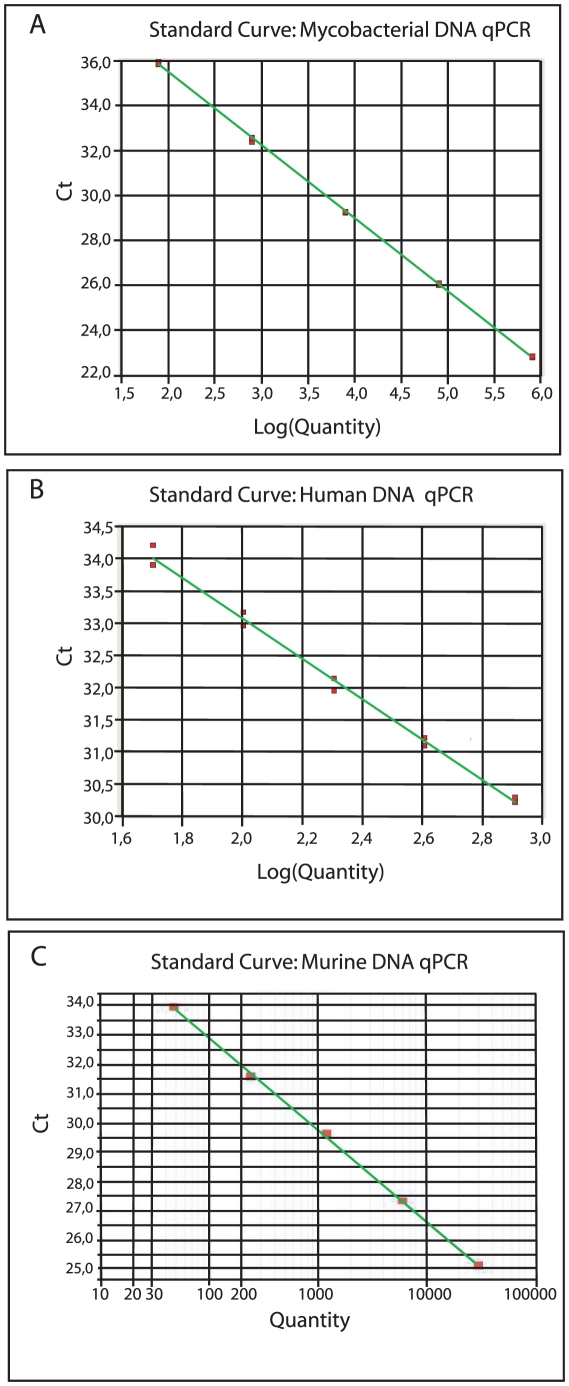
Standard Curves for the Mycobacterial, Human and Mouse targets used. Typical standard curves for the qPCR assays generated from serial dilutions of genomic *M. tuberculosis H37Rv* with slope −3,255 (A.), human genomic DNA with slope −3,141(B.) and mouse genomic DNA with slope −3,225(C.). The PCR reactions display similar efficiency (E) of near 100% as given by the equation E = 10^(−1/slope)^−1.

The intra- and inter-run variability was assessed as described by Rutledge and Cote [10] by evaluating the standard deviation in the threshold cycle (C_T_) from independent qPCR runs of replicate standard DNA dilution series. The fluorescence threshold was set appropriately and kept fixed for the given assay when analyzing data from independent qPCR. Overall, in five independent qPCR runs, the standard deviation in C_T_ of replicate amplifications for mycobacterial *GroEL2* ranged from 0.001 to 0.206 cycles, with a mean of 0.068 cycles. Assuming a near 100% qPCR efficiency (as indicated by the standard curve in Figure 1), this corresponds to inter-assay variation in quantified *GroEL2* target molecules ranging from ±0.07% to ±15.35% with an average of ±4.80%. Taking the average standard deviation in C_T_ observed in each individual run, the intra-run variation in terms of percent quantified *GroEL2* target molecules ranged from ±3.55% to ±6.25%. For the human *β-globulin* assay, the inter- and intra assay variability was also evaluated in five independent runs. In terms of quantified human *β-globulin* molecules the inter assay variability ranged from ±1.19% to ±61.78% with an average of ±16.30%, and the intra-run variation ranged form ±9.95% to ±22.55%. Variability assessment was also done for the mouse *ppia* assay used on mouse tissues. Overall, in three independent experiments, results were reproducible with inter- and intra assay variability ranging from ±0% to ±19% with an average of ±9.5%, and ±0% to ±9% (average = ±4.5%), respectively.

### Duplex qPCR assay compared to singleplex qPCR reactions

We wanted to develop a duplex qPCR for quantitative analysis of mycobacterial numbers in mammalian host cells and tissues (human or mice). The simultaneous quantification of host DNA is advantageous both as a control for PCR inhibitors when quantifying mycobacteria, and for exact determination of the number of host cells in a sample. This is of special interest in research settings like macrophage virulence assays or for anti-mycobacterial drug testing in cell cultures where there is a need to normalize the mycobacterial growth relative to the number of host cells present in different experimental conditions.

The duplex qPCR was set up using a commercially available pre-optimized master mix for multiplexing. The performance of the primer-probe sets used was tested in individual singleplex reactions before combining them in a duplex format to ensure that the individual singleplex PCR reactions performed optimally. By evaluating the efficiency of the individual PCR reactions, as judged by the slope of the standard curves for each singleplex target, we established that the reaction conditions were optimal as all PCR reactions displayed similar and near 100% efficiency (Figure 1). We next confirmed that the duplex format worked equally well as the individual singleplex qPCR assays by comparing the performance of the duplex qPCR with the singleplex PCR reactions in three simulated situations for the human assay (Table 2): 1) Excess of human DNA, 2) Near equal amount of human and mycobacterial DNA, and 3) Excess of mycobacterial DNA. Thus, the duplex qPCR assay is robust with respect to varying ratios of bacterial to host DNA.

**Table 2:** Comparison of Duplex qPCR with corresponding Singleplex qPCR in three simulated situations.

1. Excess human DNA		
Approximate No. of Targets	Duplex qPCR Mean C_T_ (±SD)	Singleplex qPCR Mean C_T_ (±SD)
Human:12120	**24.90 (±0.01)**	**25.02 (±0.05)**
Mycobacterial:78	**36.18 (±0.02)**	**35.91(±0.07)**

2. Near equal amount of human and mycobacterial DNA		
Approximate No. of Targets	Duplex qPCR Mean C_T_ (±SD)	Singleplex qPCR Mean C_T_ (±SD)
Human:808	**29.06 (±0.09)**	**28.91(±0.06)**
Mycobacterial:788	**32.62 (±0.09)**	**32.50 (±0.13)**

3. Excess mycobacterial DNA		
Approximate No. of Targets	Duplex qPCR Mean C_T_ (±SD)	Singleplex qPCR Mean C_T_ (±SD)
Human:80	**32.33 (±0.35)**	**32.22(±0.05)**
Mycobacterial:78800	**26.12 (±0.01)**	**26.08(±0.06)**

C_T_ = Treshold cycle.

### No interference of HIV infection on the correlation of mycobacterial qPCR and CFU in infected macrophage cell cultures

It is biologically plausible that HIV components may interfere with the qPCR assay, especially the viral reverse transcriptase or protease as we used a simple DNA extraction method that resulted in crude cell lysate without further purification. To formally rule out this possibility, an initial dot plot analysis of mycobacterial qPCR versus CFU stratified for HIV infection was done. Similar linear correlations were found for qPCR and CFU in HIV negative and HIV infected macrophage cell cultures (data not shown). For the subsequent analysis, the data from HIV negative and HIV infected cultures were pooled for each of the three mycobacteria tested.

### A strong linear association between qPCR and CFU in infected macrophage cell cultures allows estimation of CFU from qPCR data

**Figure 2:**
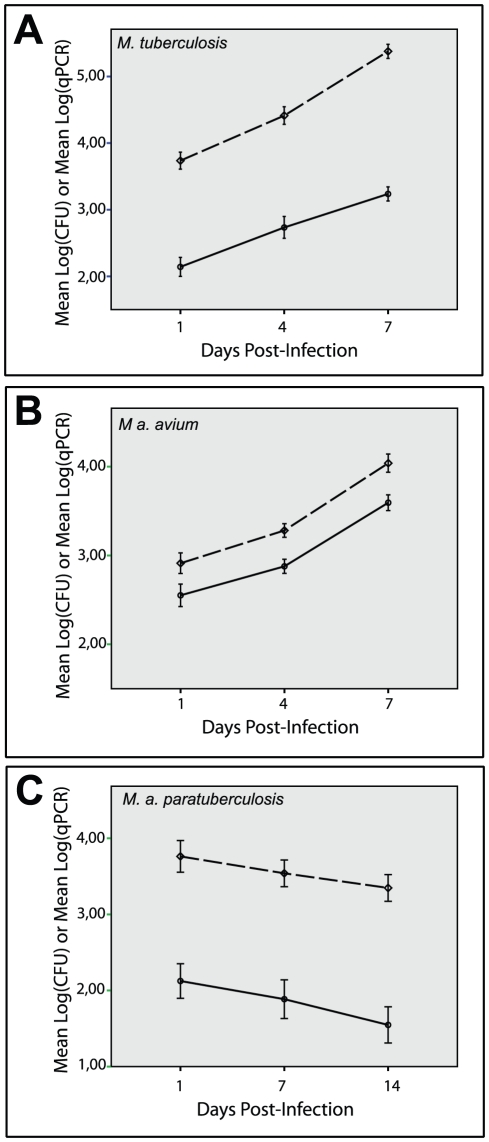
Mycobacterial growth in infected Macrophages as measured by colony counting and qPCR. Growth of *M. tuberculosis* (A), *M. a. avium* (B), and *M. a. paratuberculosis* (C) in *in vitro* infected human macrophages as monitored over time by colony counting (solid line) and qPCR (dashed line). Error bars represent 95% confidence intervals.

The conventional colony counting method is a measure of live bacteria, while the qPCR assay measures genomic load, which estimates the total number of bacteria, dead or alive, with intact DNA. To directly compare the two methods for bacterial quantification, growth of *M. tuberculosis*, *M. a. avium*, and *M. a. paratuberculosis* in infected human macrophages was monitored over time by colony counting and by qPCR. As shown in Figure 2, colony counts gave values consistently below the qPCR counts. Overall, both methods generated similar, and almost parallel, growth curves for the three mycobacteria tested. This suggests a strong linear correlation between the two methods. Notably, the geometric mean CFU to qPCR ratio remained relatively stable during the culture period for a given species: *M. tuberculosis* = 0.02; *M. a. avium* = 0.39; *M. a. paratuberculosis* = 0.02.

**Figure 3 pone-0034931-g003:**
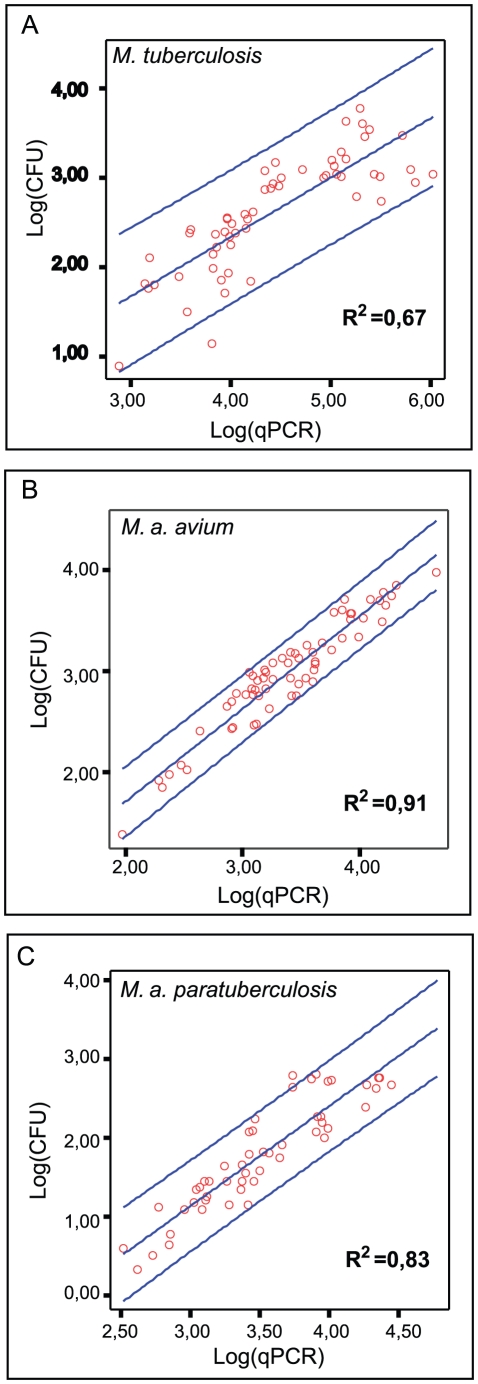
Regression models for predicting log(CFU) from log(qPCR) in macrophage cell cultures. Regression models for predicting log(CFU) from log(qPCR) for *M. tuberculosis*, *M. a. avium* and *M. a. paratuberculosis* in *in vitro* infected macrophage cell cultures derived from the data in respective training subset for each mycobacteria. Regression line in the middle with 95% prediction limits for an individual log(CFU) on each side. As it is customary to do multiple replicate CFU measurements from the same biological sample, note that the 95% prediction limits for an individual log(CFU) are wider apart than the corresponding 95% prediction limits for the predicted mean log(CFU) (not shown) for multiple measurements. Hence, the regressed point estimate for the predicted log(CFU) will be the same, but using the models as presented will tend to give wider and in fact more conservative estimates of the confidence intervals if used to predict the mean log(CFU) of multiple measurements as compared to the individual log(CFU).

To further investigate the feasibility of predicting colony counts based on qPCR results, the dataset for infected macrophage cell cultures of each mycobacterial strain was randomly split into two subsets; one training subset and one test subset. Using only the training subset for each strain, the data was analyzed for a linear association between CFU and qPCR. The number of biological culture wells with experimentally paired colony count and qPCR result is indicated by n*. A strong positive linear correlation between the log transformed paired CFU (log(CFU)) and qPCR (log(qPCR)) values was found with a Pearson correlation coefficient (r) for *M. tuberculosis* r = 0.82 (p = 5×10^−15^; n* = 58), *M. a. avium* r = 0.95 (p = 1×10^−33^; n* = 64) and *M. a. paratuberculosis* r = 0.91 (p = 1×10^−20^; n* = 51). Subsequently, a linear regression model was generated for each strain in order to enable a prediction of log(CFU) from log(qPCR). Figure 3 depicts the individual regression models for each strain along with the 95% prediction limits for an individual measurement. The regression equations are provided in Table 3. Hence, using the regression model for *M. a. avium* one may predict that for a given log(qPCR) value, for instance 3.39 (the mean), the log(CFU) will on average be 2.99 with a 95% confidence interval for an individual log(CFU) ranging from 2.66 to 3.32.

**Table 3:** Experimentally determined regression equations for prediction of log(CFU) from log(qPCR) in infected macrophage cultures.

Mycobacteria	Regression equation
*M. tuberculosis* (H37Rv)	Predicted log(CFU) = 0.662×log(qPCR)−0.311
*M. a. avium* (S72/89, serovar 4)	Predicted log(CFU) = 0.915×log(qPCR)−0.117
*M. a. paratuberculosis* (Linda)	Predicted log(CFU) = 1.266×log(qPCR)−2.661

**Figure 4 pone-0034931-g004:**
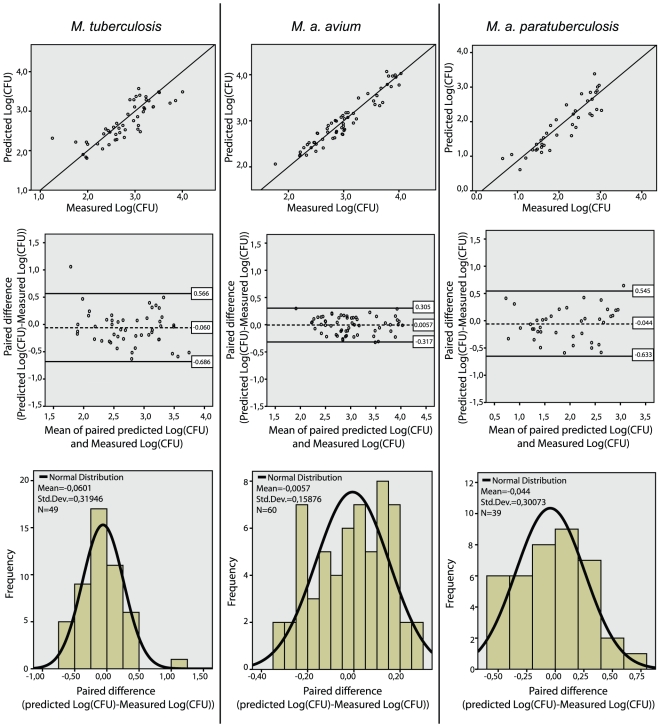
Agreement analysis of predicted log(CFU) versus actual measured log(CFU) in macrophage cell cultures. **Top panel**: Dot plot of predicted log(CFU) from qPCR data versus actual measured log(CFU) in macrophage cultures with the line of equality (black) for *M. tuberculosis*, *M. a. avium* and *M.a. paratuberculosis*, respectively. Ideally, with a perfect agreement between the two methods all point should exactly align themselves along the line of equality (y = x). **Middle panel**: Bland and Altman plot of the paired difference between the two methods against the mean value generated by the methods (i.e. the best estimate of the true value) with the mean difference (dashed line in the middle) and 95% limits of agreement (black lines). A paired difference of zero indicates perfect agreement. **Bottom panel**: Histogram of the paired difference between the two methods for the respective mycobacteria. The paired differences display a normal distribution around means which are close to zero, indicating a relative good agreement between the methods without any overt tendencies of either over- or underestimation.

As CFU and qPCR give results in different units and measure related, but biologically different entities, we used the regression models from the training subsets of each strain to predict the log(CFU) for all measured values of log(qPCR) in the test subsets. Subsequently, the agreement between the predicted log(CFU) and the paired actually measured log(CFU) was analyzed to asses the value of the regression models. The analysis was done using the Bland and Altman approach [9] and is presented graphically in Figure 4. The predictions tended to slightly underestimate the actual log(CFU). For *M. tuberculosis*, the mean difference (95% CI) between the predicted and the actual log(CFU) for an individual measurement was −0.06 (−0.69 to 0.57), while for *M. a. avium* it was −0.006 (−0.32 to 0.31) and for *M. a. paratuberculosis* it was −0.04 (−0.63 to 0.55). Expressed as the ratio of geometric means of predicted to actual CFU of an individual measurement this approximates into a mean ratio (95% CI) for *M. tuberculosis* of 0.9 (0.2 to 3.7), while for *M. a. avium* it is 1.0 (0.5 to 2.0) and for *M. a. paratuberculosis* it is 0.9 (0.2 to 3.5). Since a perfect agreement would give a ratio of exactly 1.0, the overall level of agreement seems satisfactory, especially considering the inherent variability of CFU due to mycobacterial clumping.

### Strong, but distinct time dependent linear correlations between CFU and qPCR in tissue homogenates from *M. a. avium* infected mice

Pathogenic mycobacteria are capable of persisting in immunocompetent hosts causing a chronic, latent infection. It is well established that tissue macrophages are the primary host cells for mycobacteria and initiate the innate immune response [11]. Furthermore, in mice infected by *M. tuberculosis* it has been shown that naïve T-cell activation takes place in the regional lymph nodes by day 9 post-infection and that the acquired immune response in lung tissue is established by about day 20 post-infection which is accompanied by an increased ability of the host to control *M. tuberculosis* growth [12]. A similar immune response to *M. a. avium* may be expected in infected mice. Hence, in contrast to mycobacterial infection of macrophage cell cultures, it is expected that the *in vivo* host immune response in infected animals may affect the correlation between viable bacilli counts (CFU) and total genome counts (qPCR) over time with the onset of acquired immunity. Comparing CFU counts, which reflect viable bacteria, to total genome counts of mycobacteria in animal models may thus, be useful for the understanding of the pathogenesis and host-pathogen equilibrium [3].

**Figure 5 pone-0034931-g005:**
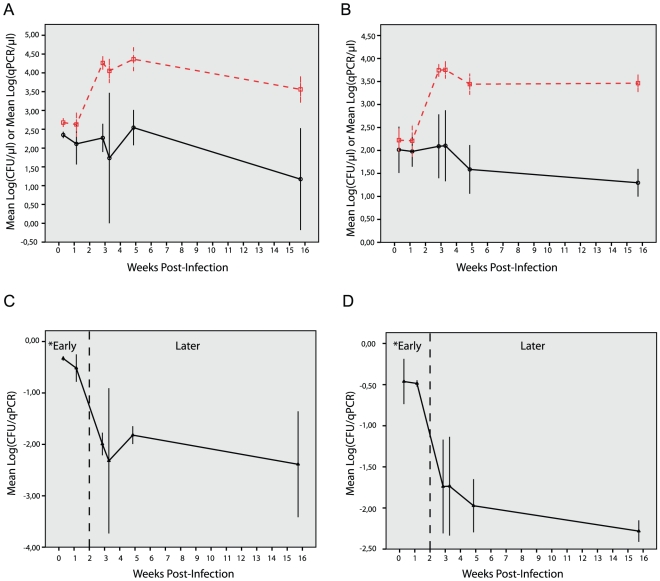
Quantification of *M.a.avium* in infected mouse tissue as measured by colony counting and qPCR. Experimentally determined quantification curves of *M. a. avium* in infected mouse spleen (A) and liver (B) as measured by viable CFU counts (solid line) and total qPCR counts (dashed line). Values are means of log(CFU) (○) or log(qPCR) (□) from 3 to 4 mice at each time point. Analysis of the mean CFU to qPCR ratio (▴) in spleen (C) and liver (D) shows that the geometric mean ratio decreases significantly between early and later time points.* p-value for difference in mean log(CFU/qPCR) for Early vs. Later time points in spleen p<0.0001 and liver p<0.0001. All error bars are ± 2×SEM.

We quantified *M.a. avium* in the spleen and liver of infected mice over time by both CFU and qPCR (Figure 5, panel A and B). As evident in Figure 5, the quantification curves by the two methods are virtually parallel and similar in shape prior to day 9 post-infection similar to the observations in macrophage cultures. The curves are also parallel from day 20 and onwards post-infection. This suggests linear but distinct correlations between CFU and qPCR during these time periods, i.e. one correlation prior to the onset of the acquired immune response (before day 9 post-infection) and a different linear correlation once the acquired immune response is established (after day 20 post-infection). However, between day 9 and day 20 post-infection there is a sharp rise in total genome counts (qPCR) indicating active mycobacterial replication while the viable counts (CFU) rises less sharply suggesting an increased ability of the host to control or kill *M. a. avium*. This observation is in line with the view that the development of the acquired immune response changes the underlying relationship of CFU and qPCR, with the consequent loss of linear correlation during the time period of immunological transition.

The CFU to qPCR ratio decreased significantly between early (day 2 and 8) versus later (day 20, 23, 34 and 110) time points in both spleen (from geometric mean ratio of 0.37 to 0.01) and liver (from geometric mean ratio of 0.34 to 0.01) with a mean fold difference in ratios (95% CI; p-value) of 48 (18–128; p<0.0001) and 26 (12–56; p<0.0001), respectively (Figure 5, panel C and D). Interestingly, in the early phase of infection the initial geometric mean ratio of CFU to qPCR in both spleen(0.37) and liver (0.34) was similar to the geometric mean ratio observed in macrophage cell cultures for *M. a.avium* (0.39). This suggests a similar correlation between CFU and qPCR count in macrophage cultures and tissue in the absence of an acquired immune response. Furthermore, the drop in mean ratio over time in infected mice coincides with the transition from the early to the later phase of infection and the onset of adaptive immunity. The decreased CFU to qPCR ratio in the later phase of infection in both tissue types suggests relatively stable pathogen-host equilibrium with low rate of bacterial replication. However, it should be noted that the qPCR method will quantify bacterial DNA and may thus overestimate the viable tissue bacterial load due to possible accumulation of dormant bacteria or mycobacterial DNA from dead bacteria over time. On the other hand, the CFU method could further underestimate the bacterial load in chronic infections as it may fail to detect dormant forms of mycobacteria persisting in the tissues that are inefficient in forming colonies on agar, but that will be enumerated by qPCR [13].

**Figure 6:**
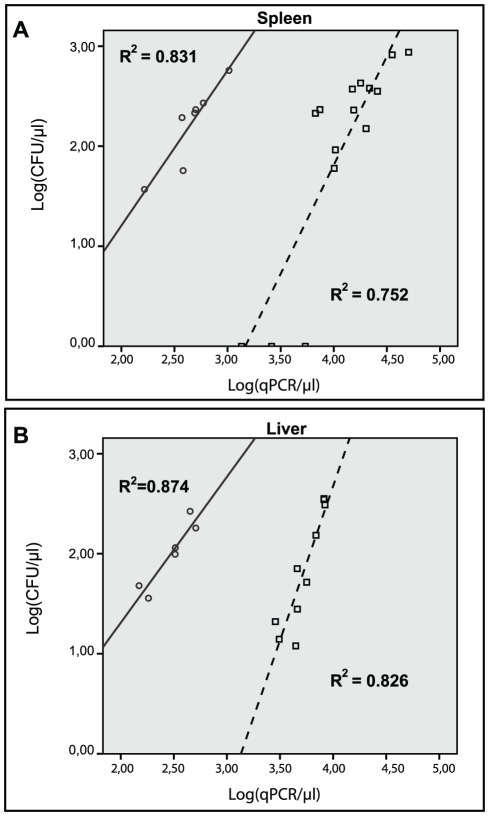
Correlation between colony counts and qPCR in *M.a.avium* infected mouse tissue. The linear relationships are similar in spleen (A) and liver (B), but distinct for the early (○, solid line) and later phase of infection (□, dashed line).

As the relationship between CFU and qPCR counts changed over time in the mouse model, we analyzed data from the early phase (<day 9 post-infection) and the later phase (≥day 20 post-infection) separately (Figure 6). The number of independent murine samples with experimentally paired colony count and qPCR result is denoted by n^#^. We found strong, but distinct positive linear correlations between log(CFU) and log(qPCR) for the early and later phase of infection in both spleen (r = 0.91; p = 0.004; n^#^ = 7 and r = 0.87; p<0.001; n^#^ = 15, respectively) and liver (r = 0.94; p = 0.006; n^#^ = 6 and r = 0.91;p<0.001; n^#^ = 10, respectively)

## Discussion

In this study we have established a rapid and reliable duplex real-time quantitative PCR method that allows the simultaneous quantification of mycobacterial and mammalian host DNA (human or mice). The duplex qPCR method proved to be robust with respect to varying ratios of bacterial to host DNA and HIV co-infection did not interfere with the performance of the assay. We used the duplex approach throughout the paper (Figure 2 through [Fig pone-0034931-g003] [Fig pone-0034931-g004] [Fig pone-0034931-g005] 6) with the simultaneous quantification of host DNA serving as a useful integral control for PCR inhibitors when quantifying mycobacteria. In the present study, we did not need to utilize the determined number of host cells to normalize mycobacterial counts. Because paired CFU and qPCR measurements were performed on samples from the same experimental well or tissue homogenate, it is appropriate to assume that the number of host cells is constant for a given pair. Hence, the actual number of measured host cells is redundant for the presented correlational analysis of the biological paired CFU to qPCR. However, the need to normalize for the number of host cells may be a prerequisite to conduct an appropriate data analysis when comparing mycobacterial counts under different experimental conditions to account for differences in growth and death of host cells. This is not normally done using CFU quantification of mycobacteria and thus represents a clear advantage of the duplex qPCR method that we have utilized in a previously published report [6].

Real-time polymerase chain reaction based quantification of DNA is generally regarded as a very sensitive and reliable technique, although the precision may decrease at very low target numbers [14], [15]. All our qPCR assays displayed optimal reaction conditions as evidenced by their efficiencies of near 100%. The precision level of the mycobacterial qPCR assay was good and within acceptable limits [10]. Although the human assay displayed higher variation, it was probably caused by the low target range used in the assay as PCR in general is especially prone to variation between replicates below 1000 targets per reaction [10].

A critical step for successful qPCR is the DNA extraction. Slow-growing mycobacteria like *M. tuberculosis* have a unique gram positive-like cell wall covered by a waxy lipid rich envelope which makes DNA extraction more difficult, but several methods for mycobacterial DNA extraction from various sources have been described (reviewed in [16] and [17]). A number of studies have advocated the usefulness and simplicity of bead beating for DNA extraction from mycobacteria [18], [19], [20], [21]. We successfully employed a rapid DNA extraction protocol based on simple boiling and mechanical bead beating for DNA extraction from infected macrophage cell cultures. However, this simple protocol proved inadequate for tissue samples yielding inconsistent results due to the presence of PCR inhibitors. Therefore, the DNA extraction had to be further optimized by including a commercial kit to ensure inhibitor-free DNA extracts from tissue samples. Some DNA loss may be inevitable during extraction resulting in underestimation of the DNA content and thus tissue bacterial load. However, the total DNA concentration (ng/g tissue) measured after extraction was quite similar for different samples of the same tissue type, but varied between tissues (higher for spleen than for liver, data not shown).

In this paper, we demonstrated that our mycobacterial qPCR assay works for *M. tuberculosis*, *M. a.avium* and *M. a.paratubeculosis*. However, we acknowledge that a wider specificity test, using DNA from other mycobacterial species, has not been performed for the reported assay. Thus, it should be noted that a standard nucleotide BLAST search (NCBI database) using the 103 base pair long mycobacterial target indicates that the assay may have limitations and will probably not work for some mycobacteria such as *M. leprae* and *M. ulcerans* due to single base pair mismatches between the target and primers or probe used.

Other studies have also described the relationship between CFU counts and qPCR counts for mycobacteria in infected cell cultures [2] and tissue [2], [3]. However, to our knowledge no study has so far analyzed whether qPCR data can be used to predict actual CFU counts. The observed CFU counts in this study were 1 to 2 orders of magnitude below the qPCR counts, which is similar to findings by Lewin et al. [2]. Like us, Lewin et al. also used a quantitative TaqMan-PCR based approach to enumerate mycobacteria in infected tissue and compared it to CFU counts. Interestingly, they found that CFU counts of bacilli Calmette-Guèrin (BCG) were 1–2 log below the qPCR counts in tissues from infected guinea pigs. There may be several explanations for this ranging from biological to technological. Bacterial aggregation would probably underestimate the number of viable bacteria quantified by colony counting whereas qPCR, on the other hand, cannot discriminate dead from live bacteria. It is also plausible that qPCR, which is based on DNA amplification, is a technologically more sensitive method as compared to colony counting. Thus, keeping in mind that CFU and qPCR measure related, but biologically distinct entities, a lack of perfect agreement between the observed CFU counts and the CFU predicted from qPCR data is not surprising. The overall agreement between CFU and qPCR observed for the mycobacterial strains tested in macrophage cell cultures was good, and the qPCR method represents an attractive alternative for rapid and reliable quantification of mycobacteria in such settings.

Furthermore, our results also show that there are strong linear correlations between CFU and qPCR in mouse tissue before day 9 post-infection prior to the onset of the acquired immune response similar to the observations in macrophage cultures and also from day 20 post-infection and onwards when the acquired immune response is established. However, during the development of the acquired immune response between day 9 and day 20, there is a sharp rise in total DNA counts (qPCR) indicating active mycobacterial replication while the viable counts (CFU) rises less sharply suggesting an increased ability of the host to control or kill mycobacteria. This observation is also reflected in the steep fall in the ratio of viable to total bacteria between day 9 and day 20 post mycobacterial infection (Figure 5, panels C and D). Hence, it seems that *in vivo* the host immune response may modulate the underlying relationship between total mycobacterial counts and live mycobacterial counts over time and thus affect the correlation between CFU and qPCR. Therefore, isolated interpretation of qPCR data in an animal model must be done with caution; keeping in mind that qPCR represents a total count of both live and dead bacteria and that the proportion of viable bacteria may vary over time.

To conclude, we have shown that the technical difficulties of enumerating mycobacteria by traditional colony counts in eukaryotic cell cultures and tissue may be overcome by using qPCR. The qPCR method provides results rapidly which is especially useful in many research settings and circumvents the problems caused by bacterial aggregates. It has the added benefit of a wide dynamic range and offers a more practical and safer assay setup than the CFU method. Also, our analysis shows that the qPCR method may replace CFU for measurement of mycobacterial growth in eukaryotic cell cultures and it represents a valuable tool for enumerating mycobacteria in animal models.
